# Health literacy profiles in kidney transplanted patients: A cluster analysis

**DOI:** 10.1111/jorc.12515

**Published:** 2024-11-11

**Authors:** Tone Karine Vidnes, Astrid Klopstad Wahl, Marie Hamilton Larsen, Käthe Birgitte Meyer, Åsmund Hermansen, Marit Helen Andersen

**Affiliations:** ^1^ Department of Transplantation Oslo University Hospital Oslo Norway; ^2^ Department of Interdisciplinary Health Sciences University of Oslo Oslo Norway; ^3^ Department for Postgraduate Studies Lovisenberg Diaconal University College Oslo Norway; ^4^ Department of Social Work, Faculty of Social Sciences, Child Welfare and Social Policy Oslo Metropolitan University Oslo Norway

**Keywords:** cluster analysis, health literacy, kidney transplantation, profiles

## Abstract

**Background:**

Health literacy is important in chronic conditions, such as kidney transplantation. Understanding patients' health literacy profiles can assist tailoring follow‐up and educational programmes to the health literacy needs of vulnerable kidney transplant recipients. This approach enabled us to cluster patients according to their profiles of challenges and strengths in different health literacy domains.

**Objectives:**

This study aimed to identify different health literacy profiles within kidney transplant recipients and what characterized the different profiles.

**Design:**

Cross‐sectional study.

**Participants:**

One hundred ninety‐five kidney transplanted recipients were included.

**Measurements:**

We used the self‐reported Health Literacy Questionnaire and analyzed using Ward's method (hierarchical cluster approach). We also collected background characteristics and clinical variables, including psychological distress (Hopkins Symptoms Checklist) and perceived health status (visual analogue scale, EuroQol‐5D).

**Results:**

The analysis revealed four clusters with substantial differences in health literacy profiles. One cluster's patients had the most challenges in all health literacy domains constituting 24% of the sample. Compared to the other three clusters, this cluster was associated with shorter duration of kidney disease, higher number of patients in dialysis before transplantation, higher percentage of male patients, lower number of kidneys from living donors, higher number of patients not working and higher representation of psychological distress. All four clusters reported the most challenges in the same domain: the ability to critically appraise health information.

**Conclusion:**

In kidney transplant recipients, profiling clusters with the Health Literacy Questionnaire and Ward's method aids in identifying health literacy needs in vulnerable groups, enabling transplant professionals to offer tailored health literacy support.

## INTRODUCTION

Kidney transplantation is considered the optimal treatment for patients with chronic kidney disease (CKD) and the only treatment that does not involve lifelong dialysis (Von Der Lippe et al., 2014). The societal cost of dialysis is higher than the cost of kidney transplantation, including medical treatment and lifelong follow‐up (Tonelli et al., 2011) and the symptom burden from dialysis can reduce patients' quality of life (Von Der Lippe et al., 2014). After a kidney transplantation, the long‐term survival of the graft relies on patients' ability to follow routines regarding medication and lifestyle aspects, including nutrition, skincare, exercise and follow‐up (Denhaerynck et al., 2007; Jamieson et al., 2016). The definition of health literacy (HL) encompasses many necessary competencies (Nutbeam, 1986): ‘to access, understand, appraise, remember and use information about health and healthcare, for the health and well‐being of themselves and those around them’ (World Health Organization, 2022b, p. 14). These competencies are of importance managing a chronic condition such as being a kidney transplant recipient (KTR) (Van der Heide et al., 2018). Challenges in HL are associated with severe implications for health outcomes (Taylor et al., 2017). Several of the diseases that may lead to CKD (e.g., high blood pressure and diabetes), can be prevented or managed through lifestyle changes (World Health Organization, 2022b).

## LITERATURE REVIEW

There is a causal relationship between reduced HL and increased visits to emergency departments, missing dialysis treatment, reduced access to kidney transplantation and mortality (Miller‐Matero et al., 2016; Taylor et al., 2017, 2019). In a Norwegian cohort study measuring KTRs' HL with the multidimensional Health Literacy Questionnaire (HLQ), HL was reported to increase in most domains measured after the first 8 weeks after transplantation but flatten out between 6 and 12 months. The increase in HL at 8 weeks was explained by the close follow‐up provided at the transplantation centre during the first 2 months. Further, the study identified a vulnerable phase for KTRs at 6 months, with decreasing HL in two domains capturing social support for health and managing their own health, explained by the return to their home context and more frequent follow‐up by healthcare personnel (Dahl et al., 2021). However, research identifying who are at risk of HL challenges in the KTR population is lacking. Psychosocial issues (e.g., depression and anxiety) can also arise, with as many as 30% of KTRs experiencing mild to severe depression after transplantation (De Pasquale et al., 2020).

Healthcare and patient information are often based on a one‐size‐fits‐all strategy, and revealing HL patterns or clusters in the KTR population may indicate which patient groups will need an individual strategy in certain HL areas. Because patients with different HL challenges have different needs according to context and individual resources, it is important to know their individual HL patterns to tailor their follow‐up (Bakker et al., 2021; Larsen et al., 2021)—especially regarding patient education. Kidney transplantation is expensive (Åsberg et al., 2020), so it is crucial for patients to follow healthcare professionals' advice on how to care for the graft to ensure the success of the procedure (Jamieson et al., 2016). If patients are not capable of making good decisions, profiling their HL strengths and challenges can aid in structured health information provision and follow‐up.

This study aimed to identify and characterize recent KTRs' HL profiles.

## MATERIALS AND METHODS

Our single‐centre study was based on baseline data collected in the clinical trial ‘Knowledge MAnagement for renal transplanted Patients’ (KnowMAP), which tested the effect of a health communication intervention among 195 KTRs. Consort statement and flow diagram are published elsewhere (Vidnes et al., 2024). The study was conducted at Oslo University Hospital, the only transplant centre in Norway. Data were collected 2–5 days posttransplantation, prior to the randomization in the main study, and before the education programme and regular follow‐up care started.

We used the HLQ to measure HL (Osborne et al., 2013) (Table 1), the EuroQol‐5D (EQ‐5D)'s visual analogue scale (VAS) to measure subjective health status (Garratt et al., 2021; Rabin & Charro, 2001). The Hopkins Symptoms Checklist (HSCL‐5) was used to measure psychological distress (Schmalbach et al., 2021; Strand et al., 2003; Tambs & Moum, 1993). All measurements were self‐reported. Clinical variables included duration of kidney disease, dialysis treatment, time on dialysis and transplantation from a living or deceased donor, also self‐reported. Demographic variables included age, gender, education, marital status and employment status (working, student, unemployed or disabled/retired) (Table 2).

**Table 1:** Health Literacy Questionnaire.

Scales	Number of items	Response scale
Part 1		
1. Feeling understood and supported by healthcare providers	4	1 = *strongly disagree*; 2 = *disagree*; 3 = *agree*; 4 = *strongly agree*
2. Having sufficient information to manage my health	4	
3. Actively managing my health	5	
4. Social support for health	5	
5. Appraisal of health information	5	
Part 2		
6. Ability to actively engage with healthcare providers	5	1 = *cannot do*; 2 = *very difficult*; 3 = *quite difficult*; 4 = *quite easy*; 5 = *always easy*
7. Navigating the healthcare system	6	
8. Ability to find good health information	5	
9. Understanding health information well enough to know what to do	5	

**Table 2:** Demographic and clinical variables of the four HL clusters and total sample.

	Cluster 1 (*n* = 47)	Cluster 2 (*n* = 75)	Cluster 3 (*n* = 41)	Cluster 4 (*n* = 32)	All = 195
Age—mean (SD)	56 (13)	56.8 (14)	49.8 (13)	50.3 (14)	54 (14) (min 18, max 80)
Gender (% male)	29 (62)	47 (63)	24 (58.5)	14 (44)	114 (58.5)
Duration of kidney disease (year) (SD)	12 (10.4)	16 (14.7)	18.6 (15)	18.5 (17.3)	16 (14.5)
Receiving dialysis treatment (%)	39 (84.8)	50 (66.7)	25 (62.5)	23 (74)	137 (71.4)
Time in dialysis (weeks) (SD)	105 (91)	106 (71)	78 (77)	123 (134)	103 (90)
Education (university/college) (%)	20 (42.6)	33 (44)	19 (46)	21 (66)	93 (48)
Living alone (%)	7 (15)	10 (13)	7 (17)	7 (22)	31 (16)
Work (%)	12 (25.5)	23 (31.5)	25 (61)	14 (44)	74 (38)
Retired or disabled (%)	28 (60)	40 (55)	11 (27)	9 (28)	88 (45.6)
Kidney school before transplantation (%)	29 (63)	43 (57)	21 (51)	11 (35.5)	104 (54)
Living kidney donor (%)	11 (23)	28 (37)	19 (46)	15 (47)	73 (37)
Perceived health status (VAS from EQ‐5D) (SD)	52 (18)	59 (17.6)	59.8 (22)	54.7 (19.4)	56.8 (19)
Hopkins mean (SD)	2.1 (0.81)	1.81 (0.82)	1.67 (0.72)	1.86 (0.82)	1.85 (0.8)

*Note*: Demographic and clinical variables presented in mean and SD or number of patients with percentage.

Abbreviations: EQ‐5D, EuroQol‐5D; HL, health literacy; SD, standard deviation; VAS, visual analogue scale.

All eligible patients from 11 March 2020 to 17 June 2021 were provided oral and written information by the nurses at the transplant ward. All patients gave written consent before study inclusion, and completed the questionnaires on paper.

- Inclusion criteria: kidney transplanted patient ≥ 18 years
- Exclusion criteria: patients who received a kidney transplant in combination with other organs and those with insufficient proficiency in Norwegian to complete the questionnaires

### HLQ

The Australian‐developed HLQ was used to measure the multidimensional concept of HL in populations (Osborne et al., 2013). It has been validated in a Norwegian context, showing good psychometric properties, including a Cronbach's *α* of 0.72–0.81 (Urstad et al., 2020). The instrument measures nine domains to capture various HL aspects (Table 1). High scores indicate HL strengths, whereas low scores indicate HL challenges. Domains 1–5 (HLQ part 1) measure level of agreement with a Likert scale from 1 (strongly disagree) to 4 (strongly agree), with a maximum mean score of 4. Domains 6–9 (HLQ part 2) measure capabilities with a Likert scale from 1 (always difficult) to 5 (always easy), with a maximum mean score of 5. As a multidimensional concept, the HLQ has no total HL score but nine domain scores, since one can have strengths in one domain and difficulties in another (Osborne et al., 2013).

### EQ‐5D

Health status was assessed using the validated EQ‐5D questionnaire, which measures self‐reported health status with five questions and a total score of self‐perceived health with a VAS scale from 0 to 100: the higher the score, the better one's perceived health (Rabin & Charro, 2001). The questionnaire was validated in Norwegian, showing good psychometric properties (Garratt et al., 2021).

### HSCL‐5

The short version of the HSCL‐5, with five questions measuring psychological distress, was used to assess symptoms of anxiety and depression (Schmalbach et al., 2021; Tambs & Moum, 1993). The questions have four response options: ‘not’, ‘a little bit’, ‘quite’ and ‘very much’ bothered (values from 1 to 4), giving one point for each degree and a total score assessing the patient's psychological distress (Tambs & Moum, 1993). A mean value of ≥2.0 (cut‐off = 2.0) indicates psychological distress. The HSCL‐5 has demonstrated satisfactory validity and reliability when used in a variety of Norwegian populations (Schmalbach et al., 2021; Strand et al., 2003).

### Statistical analysis

We used baseline data from the clinical trial to perform cluster analysis. Descriptive statistics analyzed the population characteristics. For all HLQ domains, assumptions of normal distribution were met. Ward's method was employed to cluster data, enabling us to identify different HL patterns in the KTR population. The cluster analysis grouped the patients with the least variation in mean HLQ domains (Batterham et al., 2016). All patients completed a satisfactory number of questions at baseline, with no missing data. No imputation was necessary. We measured cluster adequacy with Ward's method by evaluating distances between cluster centroids, measuring cohesion. The different distances produced different cluster solutions (Landau & Chis Ster, 2010). The senior researchers in the group, with extensive experience in HL research and cluster analysis in HLQ decided the optimal amount of clusters together (Å. H., M. H. A. and A. K. W.). Clusters are presented as means (standard deviation [SD]) for each domain score in each cluster and accompanied by information about sociodemographic distributions across the clusters. The number of clusters were chosen by minimizing the variance within each scale and each cluster, as demonstrated in earlier HLQ research (Batterham et al., 2014). An SD of greater than 0.6 for one or more of the scales indicates that there might be significant subgroups within the cluster (Landau & Chis Ster, 2010). The method also ensures that the clusters represent different patterns of strengths and challenges in the nine HLQ domains.

The cluster analysis was performed in StataCorp (2023) and Microsoft Excel 2016. A simple linear regression analysis was then performed with baseline data from the clinical trial, investigating each cluster's sociodemographic profile and associations with HLQ domains.

### Ethical considerations

The Helsinki convention was followed throughout the entire research process. Human ethics approval was obtained by the hospitals data protection board. All participants were informed with both verbally and written information. Informed consent was gathered before inclusion in the study. Anonymity was obtained by using numerous codes in the sensitive data system.

## RESULTS

A total of 241 KTRs were invited to participate in the study; 41 did not wish to participate. Of the 200 patients included in the study, five were excluded before answering the baseline questionnaires due to dementia, postoperative complications and problems filling out the questionnaire. In the remaining 195 patients, the majority were males (58.5%), their mean age was 54 years and they had a kidney disease duration of 16 years (SD 14.5). In total, 37% received a kidney from a living donor, 137 patients (71.4%) received dialysis treatment at the time of the transplantation and the average time on dialysis was 103 weeks (SD 90). Forty‐eight percent had college‐ or university‐level education, and 38% were working at the time of the transplantation. Their mean perceived health status (VAS from 1 to 100) was 56.8 (SD 19) and mean Hopkins score was 1.85 (SD 0.8) (Table 2). Based on cluster size and HL patterns, four clusters were selected for diversity (Batterham et al., 2014). Each cluster contained 32–75 patients.

**Figure 1:**
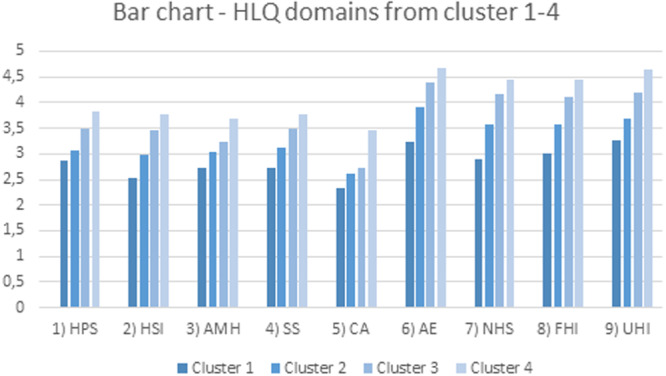
Bar chart of Health Literacy Questionnaire (HLQ) domains from 1 to 4.

Figure 1 presents clusters 1–4, with bar charts for the nine HLQ domains. The cluster with the most challenges is the lower bar chart and the cluster with the most strengths is the higher bar chart.

### Cluster 1

Cluster 1 comprised 24% of the sample (47 patients) and presented the most HL challenges compared to the total sample: reporting lower scores in five HL domains compared to the other clusters (Table 3). The mean values in domains 1–5 ranged from 2.34 to 2.87 (possible score from 1 to 4) and domains 6–9 had mean values from 2.9 to 3.25 (possible score from 1 to 5), demonstrating more challenges than the average patient group in all nine domains. The lowest mean value was Domain 5 (appraise health information), with a mean value of 2.34 and the highest value was Domain 9 (ability to understand health information well enough to know what to do), with a mean value of 3.25.

**Table 3:** Mean HLQ scale scores for the four clusters and total sample.

Patients in each cluster (*N* = 195)	Feeling understood and supported by healthcare providers (HPS)	Having sufficient information to manage my health (HSI)	Actively managing my health (AMH)	Social support for health (SS)	Appraisal of health information (CA)	Ability to actively engage with healthcare providers (AE)	Navigating the healthcare system (NHS)	Ability to find good health information (FHI)	Understanding health information well enough to know what to do (UHI)
47 (24.1%)	2.87	2.53	2.73	2.72	2.34	3.23	2.90	3.00	3.25
75 (38.5%)	3.07	2.99	3.04	3.13	2.62	3.91	3.58	3.56	3.68
41 (21%)	3.49	3.45	3.24	3.49	2.73	4.39	4.16	4.10	4.19
32 (16.4%)	3.82	3.78	3.68	3.78	3.47	4.66	4.44	4.44	4.64
195 (100%)	3.23	3.11	3.11	3.21	2.72	3.97	3.68	3.68	3.84

*Note*: Data on patients presented in numbers and percentage. All HLQ scores presented in mean.

Abbreviation: HLQ, Health Literacy Questionnaire.

Patients in cluster 1 had a higher mean age (56 years) compared to clusters 3 and 4, and a considerably higher percentage of male patients (62%) than cluster 4 (44%). Patients in cluster 1 had a shorter kidney disease duration than the other clusters, with an average of 12 years. Further, cluster 1 had a high percentage of patients receiving dialyses at the time of transplantation (84.8%). However, the mean time on dialysis treatment was the same as the average time of 105 weeks. Only 25.5% in cluster 1 were employed at the time of transplantation, and 60% were retired. Cluster 1 also had the most patients attending a local comprehensive education programme for people learning to manage and live with kidney disease (‘kidney school’) before transplantation (63%). Compared to the other clusters, fewer patients in cluster 1 received a kidney from a living donor (23%). It was also the only cluster with a higher mean Hopkins score of 2.1, thus the only cluster showing psychological distress (mean value ≥ 2.0).

### Cluster 2

Cluster 2 was the largest cluster, comprising 38% of the sample (75 patients). The patients in this cluster reported lower mean scores in all nine domains compared to the average mean score of the entire sample. The highest mean score (3.91) was in Domain 6 (ability to actively engage with healthcare providers) and the lowest was in Domain 5 (appraisal of health information), with a mean score of 2.62.

Cluster 2 had the highest mean age of the total sample (56.8) and 55% were retired. The cluster presented the second highest perceived health status (59, out of a maximum value of 100). The cluster's demographic and clinical variables were otherwise comparable to the total sample (Table 2).

### Cluster 3

Cluster 3 included 41 KTRs, representing 21% of the total sample. Domains 1–5 represented mean values from 2.73 to 3.49. Respectively, domains 6–9 had values between 4.10 and 4.39, demonstrating strengths in all domains, with the highest score in Domain 6 (ability to actively engage with healthcare providers). The lowest mean score of this cluster (2.73), was Domain 5 (appraisal of health information) and quite similar to the mean average of the study population (2.72). All the domains' mean values were high, and the patients had HL strengths in all domains except Domain 5 (Table 3).

Regression analysis of the different background variables showed that age seemed to have an impact, as cluster 3 had the lowest mean age (49.8). Further, this cluster also had the highest percentage of employed patients (61%, compared to the average of 38% in the total sample). Cluster 3 represented the lowest Hopkins score, with a 1.67 average, and the highest perceived health status of all clusters (59.8).

### Cluster 4

Cluster 4 contained 32 KTRs (16% of the sample) and had the highest mean HL scores overall, in all HLQ domains. Domains 1–5 had mean scores from 3.47 to 3.82 and domains 6–9 had mean scores from 4.44 to 4.66. The lowest mean score within this group was Domain 5 (appraisal of health information), with a mean score of 3.47 (0.75 points higher than the average mean score of the total sample). It was the only cluster not classified as having challenges in this domain.

Descriptive statistics of demographic variables in cluster 4 show that there were fewer men than average in this group (44%) and that 66% had higher education (college or university). The cluster had a lower percentage of retired patients (28%) and a high percentage of patients who had received a kidney from a living donor (47%). Surprisingly, the cluster had the lowest percentage of patients who had attended kidney school before transplantation, with only 35.5%, compared to the average of 54%. The cluster also had a longer duration on dialysis than the other clusters, with a mean of 123 weeks, but an SD of 133 weeks—with the lowest treatment time of 13 weeks and the longest duration of 598 weeks.

## DISCUSSION

Our study revealed four HL clusters, of which one deviated by having challenges in five out of nine HLQ domains (cluster 1). These domains included Domain 5 (CA), 6 (AE), 7 (NHS), 8 (FHI) and 9 (UHI). With the exception of cluster 4, all clusters encountered difficulties in Domain 5 (CA) and scored their lowest score in this domain. This suggests that the appraisal of health information may be the most challenging domain in this cohort of KTRs. Our findings support recent research (Andersen et al., 2024). A possible explanation for this particular cluster to score lower in this domain might have been due to demographic factors, such as higher mean age, living alone and a lower proportion of living donor recipients. In general, being an older male, living alone and possibly retired, having only had CKD for a short duration and undergoing dialysis before transplantation may correlate with HL challenges. Other studies report similar findings (Dahl et al., 2021; Larsen et al., 2021). However, though prior studies report that identifying as female is frequently associated with HL challenges, we did not find this (Beauchamp et al., 2015; Stømer et al., 2019). Tailored support and follow‐up care for these individuals after transplantation may therefore prove beneficial (Larsen et al., 2022; Shao et al., 2023).

All clusters scored highest on Domain 6 (AE). One explanation may be that the population awaiting kidney transplantation is carefully followed up by a nephrologist and/or kidney nurse. The results may reflect a close relationship between care providers and patients.

The most intriguing aspect of the cluster reporting the most HL challenges is that these patients had the highest level of participation in kidney school before their transplantation. Kidney school is the local hospital's education programme, aiming to strengthen patients in terms of their CKD, but also prepare for transplantation. The patients are recruited by their local nephrologist or kidney nurse, but attendance is optional. One might assume that kidney schools would have a positive impact on patients' HL, and studies indicate that education programmes are beneficial for patients (Taylor et al., 2017; Tom & Phang, 2022; Urstad et al., 2012). However, our findings indicate otherwise. A recent kidney disease diagnosis may have affected patients' ability to retain the information presented during kidney school (Jones et al., 2016). However, cluster 4—which had the most HL strengths—had the lowest percentage of participation in kidney school, but the longest duration of kidney disease. Perhaps this had an impact on their level of readiness to acquire knowledge compared to the other clusters. Additionally, they may have accessed alternative knowledge sources. In Norway, each hospital has developed educational programmes for patients with CKD. A lack of national standards for kidney schools makes it difficult to draw conclusions. Nevertheless, our findings raise the question of whether CKD patients might benefit from a national, standardized educational programme.

Globally, there is a risk of inequity in treating patients with chronic diseases (Osborne et al., 2022; World Health Organization, 2022a). It is therefore crucial to identify and categorize at‐risk patients based on their HL levels (Aaby et al., 2020; Larsen et al., 2021; Stømer et al., 2019), including in kidney care. Profiling patients in clusters aids in identifying risks and assets in the KTR population, providing clinicians with essential information about possible HL challenges following kidney transplantation (Landau & Chis Ster, 2010). In this study, the lowest scores in all clusters were for Domain 5 (CA) demonstrating difficulties with appraising health information. Our research supports previous studies using the HLQ that report Domain 5 (CA) as the most challenging (Beauchamp et al., 2015; Dahl et al., 2020; Stømer et al., 2019). Patients have access to a considerable amount of health information, and healthcare professionals communicate information in diverse ways. Unfortunately, the transfer of knowledge and information between healthcare personnel and patients is underexplored (Wahl et al., 2022). Our results indicate that KTRs faced challenges in the same HLQ domains—Domain 5 (CA), 7 (NHS) and 8 (FHI)—as patients with other chronic conditions; this finding is supported by numerous HL studies adopting a cluster analysis approach, indicating greater difficulties for patients within these HL areas (Andersen et al., 2024; Bakker et al., 2021; Dahl et al., 2021; Larsen et al., 2021).

## IMPLICATIONS FOR CLNICAL PRACTICE

Collecting information about patients who share similar characteristics provides valuable insights for transplant professionals. This study offers useful profiles to personalize interventions for KTR patients (Larsen et al., 2022; Shao et al., 2023; Shnaigat et al., 2021), identifying those who may be susceptible to HL challenges after transplantation. HL is of great importance for patients' health outcomes (Taylor et al., 2017), including those going through a kidney transplantation. Knowing which patient profiles are at risk of reduced HL is important, to guide transplant personnel in allocating extra resources to strengthen HL for specific patients. Earlier research conducted on chronic diseases, including psoriasis and rheumatic conditions, has yielded important results in the form of distinct HL profiles and clusters, which can aid in the development of targeted, effective interventions (Bakker et al., 2021; Larsen et al., 2021). We regard this strategy to be decisive for optimal treatment outcomes. Nevertheless, tailoring the follow‐up of patients with the most strengths and knowing their HL profiles may help conserve resources, enabling the most effort to be given to those who most need it. This strategy can also help meet the demands of a more efficient healthcare system, which requires timesaving follow‐up and shorter hospital stays.

### Strengths and limitations

A strength of the study is its representative sample. From March 2020 to June 2021, 195 KTRs were included as part of a larger ongoing clinical trial in Norway. Recruitment occurred during the COVID‐19 pandemic, and the number of kidneys received from living donors was higher than normal during this period (37%) (Reisæter, 2020). Otherwise, the patients represented a normal distribution of the KTR population. The sample size was determined via power calculation for the clinical trial, but no set rule governs how many participants are needed for cluster analysis. Studies show that stable results can be achieved with a similar number of people when using the HLQ questionnaire to cluster HL (Anwar et al., 2021). Another strength of the study is its use of validated questionnaires (Garratt et al., 2021; Osborne et al., 2013; Strand et al., 2003; Urstad et al., 2020). A missing imputation strategy in the HLQ was unneeded, and only three had missing answers in the HSCL‐5 and EQ‐5D's VAS scale questionnaires. As Norway has only one transplant centre, all patients received the same (quality of) treatment.

The inclusion of 195 KTRs from rural and urban areas in Norway enhances the study's generalizability. However, the increasing prevalence of KTRs in Norway with a native language other than Norwegian, and the exclusion of most of these patients from the present study poses a limitation regarding generalizability.

## CONCLUSION

In KTRs, profiling clusters with the HLQ and Ward's method aids in identifying HL needs in vulnerable groups, enabling transplant professionals to offer tailored HL support.

## AUTHOR CONTRIBUTIONS

Marit Helen Andersen and Astrid Klopstad Wahl contributed to the research idea and study design. Tone Karine Vidnes, Marie Hamilton Larsen and Åsmund Hermansen contributed to the statistical analysis. All authors contributed to the data analysis/interpretation. Marit Helen Andersen and Astrid Klopstad Wahl provided supervision and mentorship. Tone Karine Vidnes was the primary author of the manuscript and prepared Tables 1 and 2 and Figure 1. Marie Hamilton Larsen prepared Table 3. All authors contributed important content for and revision of the overall work and reviewed the final manuscript.

## CONFLICT OF INTEREST STATEMENT

The authors declare no conflict of interest.

## ETHICS STATEMENT

Approval was obtained from the Norwegian Ethics Committee for Health Research (#2019/29385). The original study (KnowMAP) was registered as a clinical trial (ClinicalTrials.gov Identifier: NCT04296955). All participants provided written, informed consent.

## Data Availability

The data set used and/or analyzed during the current study may be available from the corresponding author upon reasonable request.
